# Bioengineered Human Stromal Lenticule for Recombinant Human Nerve Growth Factor Release: A Potential Biocompatible Ocular Drug Delivery System

**DOI:** 10.3389/fbioe.2022.887414

**Published:** 2022-06-23

**Authors:** Leonardo Mastropasqua, Mario Nubile, Giuseppina Acerra, Nicola Detta, Letizia Pelusi, Manuela Lanzini, Simone Mattioli, Manuela Santalucia, Laura Pietrangelo, Marcello Allegretti, Harminder S. Dua, Jodhbir S. Mehta, Assunta Pandolfi, Domitilla Mandatori

**Affiliations:** ^1^ Ophthalmology Clinic, Department of Medicine and Aging Science, “G. d’Annunzio” of Chieti-Pescara, Chieti, Italy; ^2^ Dompé Farmaceutici SpA, Via Tommaso de Amicis, Naples, Italy; ^3^ Department of Medical, Oral and Biotechnological Sciences, Center for Advanced Studies and Technology (CAST), StemTeCh Group, “G. d’Annunzio” University of Chieti-Pescara, Chieti, Italy; ^4^ Department of Medicine and Aging Sciences, Center for Advanced Studies and Technology (CAST), “G. d’Annunzio” University of Chieti-Pescara, Chieti, Italy; ^5^ Dompé Farmaceutici SpA, Via Campo di Pile, Wn, L’Aquila, Italy; ^6^ Academic Ophthalmology, Division of Clinical Neuroscience, School of Medicine, University of Nottingham, Nottingham, United Kingdom; ^7^ Tissue Engineering and Cell Group, Singapore Eye Research Institute, Corneal and External Department, Singapore National Eye Centre, Singapore, Singapore

**Keywords:** decellularization, drug delivery, microparticles, nerve growth factor, scaffold, smile, stromal lenticule, tissue engineering

## Abstract

Small incision lenticule extraction (SMILE), is a surgical procedure for the myopia correction, during which a corneal stromal lenticule is extracted. Given that we have previously demonstrated how this discarded tissue could be repurposed as a bio-scaffold for stromal engineering, this study aimed to explore its use as an ocular drug delivery system of active molecules, using neurotrophic factor Nerve Growth Factor (NGF). We employed human stromal lenticules directly collected from healthy donors undergoing SMILE. Following a sodium dodecylsulfate (SDS) treatment, decellularized lenticules were incubated with a suspension of polylactic-co-glycolic-acid (PLGA) microparticles (MPs) loaded with recombinant human NGF (rhNGF-MPs). Fluorescent MPs (Fluo-MPs) were used as control. Data demonstrated the feasibility to engineer decellularized lenticules with PLGA-MPs which remain incorporated both on the lenticules surface and in its stromal. Following their production, the *in vitro* release kinetic showed a sustained release for up to 1 month of rhNGF from MPs loaded to the lenticule. Interestingly, rhNGF was rapidly released in the first 24 h, but it was sustained up to the end of the experiment (1 month), with preservation of rhNGF activity (around 80%). Our results indicated that decellularized human stromal lenticules could represent a biocompatible, non-immunogenic natural scaffold potential useful for ocular drug delivery. Therefore, combining the advantages of tissue engineering and pharmaceutical approaches, this *in vitro* proof-of-concept study suggests the feasibility to use this scaffold to allow target release of rhNGF *in vivo* or other pharmaceutically active molecules that have potential to treat ocular diseases.

## Introduction

The cornea is a highly organized structure of the anterior segment of the eye and forms part of its protective tunic. By virtue of its transparency and refractive power it serves an important role in vision, by transmitting and focusing light rays. Several pathological conditions such as infection, inflammation, degenerations, dystrophies, and neuropathies can affect corneal transparency, shape, thickness power and sensitivity in a manner that compromises light transmission and focusing, with consequent visual impairment and/or blindness (Gonzalez-Andrades et al., 2011).

Among the various therapies, the recent introduction of nerve growth factor (NGF) eye drops has made a significant difference to the management of neurotrophic keratopathy (Sacchetti and Lambiase, 2017). NGF is a soluble protein belonging to the family of neurotrophic factors. Its topical use as recombinant human NGF (rhNGF) produced in *Escherichia Coli*, has shown promising therapeutic results for ocular disease treatment (Bonini et al., 2018; Mastropasqua et al., 2020; Sacchetti et al., 2020). However, topical administration of rhNGF poses limitations in that it requires to be transported and stored in a frozen state and needs frequent instillation that can affect compliance.

Therefore, to improve compliance and ensure a sustained release on the eye of drugs such as rhNGF, several delivery systems have been explored. For example, contact lenses or synthetic scaffolds based on hydrogel and/or collagen polymers, loaded with active molecules, have shown translational potential for replacing and regenerating diseased corneal stromal tissue (Maulvi et al., 2016; Lynch et al., 2020; Xeroudaki et al., 2020). However, these are associated with inherent limitations of irritation, blurring of vision, hypoxia, and variable bioavailability due to the physiological barriers of the eye, which limit the drug release and concentration in the ocular site.

For this reason, the development of a natural, biocompatible, non-immunogenic delivery system that ideally combines the advantages of tissue engineering and pharmaceutical technologies, may allow us to obtain effective concentrations of drug molecules in the eye for a sufficient period.

The corneal stromal lenticule provides a possible source of a bio-scaffold. The lenticule constitutes the discarded tissue derived from refractive treatments, a technology based on femtosecond lasers (FSL) that has recently introduced a new dimension in corneal surgery (Sioufi et al., 2021). With exquisite precision, during the surgical procedure known as small incision lenticule extraction (SMILE) or Refractive lens extraction (ReLex), an intra corneal stromal lenticule of desired power and shape is sculpted and removed through a small incision (Reinstein et al., 2014). Given that such tissue is generally discarded, recently it has been put to the innovative applications in the treatment of corneal stromal diseases such as keratoconus, hyperopia, and corneal perforation where it physically adds to the thickness and substance of the tissue and alters the shape (curvature) of the cornea (Pradhan et al., 2013; Mastropasqua et al., 2018; Riau et al., 2021). Although these procedures have shown effective and safe results, immune rejection and inflammation can still occur following allogenic lenticule implantation (Hashimoto et al., 2016). In this context, we have previously demonstrated that customized stromal lenticules derived from human cadaveric corneoscleral tissue, can be decellularized to reduce or eliminate the risk of immune rejection (Riau et al., 2020). Furthermore, since these lenticules also retained their transparency and native stromal architecture, we hypothesized a possible repurpose as a natural acellular bio-scaffold suitable in tissue engineering (Yam et al., 2016). Therefore, in this study, by using discarded stromal lenticules directly derived from SMILE procedures, we aimed to address the unmet need of obtaining a biocompatible, non-immunogenic delivery system that could be loaded with a desired drug for a sustained release over time. We report on the feasibility to incorporate microparticles (MPs), laden with rhNGF in decellularized human SMILE-derived stromal lenticules, and the release of such growth factor over time from the scaffold, assumingits potential use as ocular drug delivery system.

## Materials and Methods

### Ethics Statement

Patients undergoing laser refractive surgery for moderate myopia were enrolled (spherical equivalent range −4.00 to−6.00 D, with astigmatism less than 0.75 D). All patients were over the age of 18 and had a normal corneal topographic pattern and stable refraction for at least 1 year preoperatively. This study was approved by the “G. d’Annunzio” of Chieti-Pescara University’s Institutional Review Board and Ethical Committee (Approval number: 03/07-02-2019) and adhered to the tenets of the Declaration of Helsinki. After a detailed explanation of the study, written informed consent for the use of extracted lenticules was obtained from each patient before the procedure. Patients who had any ocular pathology other than refractive error or history of ocular surgery were not included.

### Surgical Technique

Corneal stromal lenticules were obtained following SMILE procedure performed, as previously described, by using the VisuMax system (Carl Zeiss Meditec, Germany) (Mastropasqua et al., 2017). Briefly, the diameter of the lenticules was set between 6.00 and 6.3 mm. The cap diameter was 7.5 mm and its thickness was 120 µm. Side cut length ranged between 3 and 3.3 mm in all cases. According to the Visumax software, a correction power ranging between −4D and −6D generated a biconvex lenticule with a minimum thickness of 30 μm at the peripheral edge and maximum thickness varying between 90 and 160 μm, at the center. The extracted stromal lenticules (*n* = 50) were transferred in sodium chloride physiological solution (0.9% NaCl) and stored at −80°C in glycerol (Liu et al., 2017) until experiments.

### Decellularization Treatment

Human corneal lenticules, having a thickness between 100 and 150 μm, were decellularized according to the following procedure. Samples were thawed, rinsed in phosphate buffer solution (PBS 1X, Sigma-Aldrich) and incubated in a 0.1% SDS solution for 24 h under 300 rpm agitation at room temperature (RT) as previously described (Yam et al., 2016). Following 3 washes in PBS 1X (24 h each), decellularized stromal lenticules were analyzed to confirm keratocytes removal and extra-cellular matrix (ECM) organization and content.

### Phalloidin and DNA Immunostaining

Immunostaining analyses (*n* = 6) were directly performed on stromal lenticules (0.1% SDS-treated or not) fixed in paraformaldehyde 4% for 10 min (Santa Cruz Biotechnology), permeabilized with 0.1% Triton (5 min; Sigma-Aldrich) and incubated for 1 h with the AlexaFluor 488-conjugated phalloidin (Invitrogen, A12379) primary antibody. The 4′,6-diamidino-2-phenylindole (DAPI; cat. D9542, Sigma-Aldrich) was used to stain the nuclei. Immunostained lenticules were finally directly mounted on coverslip and fluorescence images were acquired both in 2D and 3D (Z-stack; *x*, *y* and *z* axis) using the Zeiss LSM800 confocal microscope (Oberkochen, GE).

### DNA Extraction and Quantification

Following an overnight incubation at 56°C with proteinase K (50 μg/ml, Promega), DNA from control (CTRL) and 0.1% SDS-treated lenticules (*n* = 3) was isolated by using Phenol-Chloroform (Sigma Aldrich; 77617) and potassium acetate (3M, Sigma-Aldrich; 71196) reagents. DNA was precipitated with ethanol, dissolved in nuclease-free water and quantified by NanoDrop 2000c Spectrophotometer (ThermoFisher Scientific).

### Transmittance

The spectral transmittance of control (CTRL) and 0.1% SDS-treated lenticules was assessed using a microplate reader (SpectraMAX 190; Molecular Devices, San Jose, CA, United States). Lenticules were placed in water in 96 well plate and inserted into the spectrophotometer for transmittance measurement using a wavelength ranged from 380 to 780 nm. The transmittance coefficient (Ct) was calculated as Ct = I_t_/I_0_ (I_t_ = transmitted light intensity; I_0_ = incident light intensity). Lenticules’ Ct were normalized with the corresponding Ct of water (blank).

### Trasmission Electron Microscopy

Stromal lenticules (CTRL and 0.1% SDS-treated) were processed for Trasmission Electron Microscopy (TEM) morphology evaluation and morphometric analysis (*n* = 3). CTRL and 0.1% SDS-treated lenticules were fixed with 3.5% glutaraldehyde in 0.1 M sodium cacodylate (NaCaCO) buffer for 1 h and stored at 4°C. For embedding, samples were post-fixed in 2% OsO_4_ in the same buffer for 2 h and block-stained in uranyl acetate replacement. Ultrathin sections (∼50 nm) were cut using a Leica Ultracut R microtome (Leica Microsystem) with a Diatome diamond knife (Diatome Ltd.) and double-stained with uranyl acetate replacement and lead citrate. Sections were viewed in a FP 505 Morgagni Series 268D electron microscope (FEI Company), equipped with Megaview III digital camera and Soft Imaging System at 60 kV. For the quantitative analyses in each sample, cross-sectional views of lenticules were imaged as follows: 1) collagen fibrils were evaluated at magnification of 44.000X in 3 random fields from 3 different control and decellularized lenticules. In each micrograph the number of fibrils was quantified and expressed as number/μm^2^, and the mean fibril size was measured and reported in nm 2) Structure of collagen fibers was evaluated at magnification of 14.000X in 3 different control and decellularized lenticules. Collagen fibers were classified in highly organized, partially organized and disorganized fibers by the presence of a precise disposition of fibrils inside each fiber or a partially/completely disorganization. The number of highly organized, partially organized and disorganized fibers present in each sample was counted and reported as percentage of the total fibers.

### Extra-Cellular Matrix Histology and Quantification Analysis

Histological sections (5 μm) of control and 0.1% SDS-treated lenticules, fixed in formalin and embedded in paraffin (*n* = 6), were stained with Hematoxylin and Eosin (H&E), Alcian Blu (Bio-Optica, cat^#^ 04-160802) and Periodic Acid-Schiff (PAS; Bio-Optica, cat^#^04-130802) kits. The sections were photographed under light microscopy using 10X and 20X objectives. Lenticules thickness (5 standard position) and staining intensity of PAS and Alcian Blue were measured by using ImageJ software (NIH, United States ImageJ software, public domain available at: http://rsb.info.nih.gov/nih-image/).

### Lenticules Engineering With Fluorescent PLGA- MPs

0.1% SDS-treated lenticules (decellularized; *n* = 6) were dehydrated for 2 h at 60°C and then engineered with green, fluorescent PLGA-MPs (Fluo-MPs; 445/500 nm; Supplied by S.I.C. ^#^LGFG5000). In details, 5 mg of Fluo-MPs were suspended in 0.175 ml of 0.9% NaCl (highest saturation degree) and one lenticule per tube was incubated with MPs solutions for 3, 5, 7, and 12 h at RT with orbital shaking at 200 rpm. Lenticules were then removed from the suspension and washed 10 times in 0.4 ml of 0.9% NaCl. Finally, engineered lenticules were fixed in paraformaldehyde 4% (10 min RT). To mark out the lenticules’ upper and lower surface, an anti-Collagen I primary antibody (Abcam, Cat^#^ ab34710) in BSA 1% (Bovine Serum Albumin) was used (tissue permeabilization step was not performed in order to avoid the damage of the Fluo-MPs possibly loaded to the lenticule). Following 3 washes in PBS 1X (5 min each) lenticules were incubated in BSA 1% for 1 h with an Anti-rabbit Cy3 secondary antibody (Jackson ImmunoReasearch, Laboratories, Cat^#^ 111-165-144). Immunostained lenticules were directly mounted on coverslip and fluorescence images were acquired both in 2D and 3D (Z-stack; *x*, *y,* and *z* axis) using the Zeiss LSM800 confocal microscope (Oberkochen, GE). Fluo-MPs counts were performed using ImageJ software (NIH, United States ImageJ software, public domain available at: http://rsb.info.nih.gov/nih-image/).

### Preparation of MPs Loaded With rhNGF

PLGA- MPs loaded with rhNGF were prepared using a “customized” double emulsion with solvent evaporation method. Briefly, 300 µl aqueous solution (W1), containing 150 µl rhNGF (0.85 mg/ml) was prepared in formulation buffer (50 mM Sodium phosphate monobasic monohydrate, 100 mM Sodium Chloride, pH 7.2), 60 µl human serum albumin 0.1 g/ml (rhHSA, Merck) and 150 µl of polyethylene glycol 400 (PEG400, Merck), (ratio 1:40:1, v/w/v) was prepared. The W1 was added to 7.5 ml of the oil solution (O), containing 20% w/v of PLGA (Expansorb DLG752-2A, Merck) in ethyl acetate (Merck), and was homogenized at 17400 rpm for 3 min, by a Ultraturrax T25 digital homogenizer (IKA), using 8G dispersing tool. The obtained emulsion W1/O was quickly added to 30 ml of second aqueous solution (W2), containing 1% w/v of polyvinyl alcohol (PVA) (Mowiol 40–88, Merck) doped with 900 µL of ethyl acetate and then homogenized again at 12,000 rpm for 30 min. The resulting double emulsion (W1/O/W2) was stirred for 3 h at 500 rpm, at RT using overhead stirrer RW20D (IKA), with a PTFE-coated-2-bladed propeller stirrer, to allow organic solvent evaporation and the precipitation of MPs. The suspension was collected in 50 ml polypropylene tubes (Eppendorf LoBind protein) and centrifuged at 7,400 rpm, for 15 min, at 4°C. The MPs were isolated and washed 6 times, using 50 ml milliQ water, by centrifugation at 7,000 rpm for 15 min at 4°C. Afterwards, the MPs were re-suspended in 2% w/v trehalose (Merck) and dispensed in ISO glass type siliconized Class 1 vials to be freeze-dried. After the lyophilization process, the dried MPs was analyzed by Mastersizer 2000 (Malvern) and by Scanning Electron Microscopy to check the requested morpho-dimensional specifications of final product.

### Encapsulation Efficiency and *In Vitro* Release Kinetic Profile Evaluation of rhNGF-MPs

The encapsulation efficiency (EE; percentage of the drug substance included the MPs shell) and the *in vitro* release of the rhNGF from the sustained release system were evaluated as follow described. The EE of rhNGF-MPs (%) and the drug loading (ng rhNGF/mg MPs), was determined by ELISA assay using the kit RayBio Human beta-NGF (Raybiotech). Briefly, about 10 mg of MPs (freeze-dried powder) was treated with 1 ml of ethyl acetate to ensure the degradation of the polymeric matrix of PLGA. Then, 1 ml of aqueous phase (FB) was added to the organic phase to ensure the rhNGF extraction and the sample was centrifuged for 5 min at 7,500 rpm, at 4°C. Finally, the aqueous phase was collected and appropriately diluted with FB to further ELISA analysis. Particularly the detect range of assay kit was 16–5,000 pg/ml. The test was performed according to manufacturer instructions. To evaluate the *in vitro* release kinetics of rhNGF from MPs, about 10 mg of MPs (triplicate) were suspend in 1 ml of PBS pH 7.4 (suspending medium) in 2 ml polypropylene LoBind protein tubes (Eppendorf) and incubated at 37°C under orbital shaking (80 rpm). At every pre-established collection time points, the samples were centrifuged (7,500 rpm- 7 min −4°C) and the suspending medium was completely collected and immediately sored at −20°C. Then, the initial volume of suspending medium was completely restored with fresh PBS to re-suspend the MPs pellet. The withdrawal of samples was carried out every 2-3 days (no later than 4 days) to better preserve the physico-chemical and biological characteristics of rhNGF. The samples, collected during the release kinetics test, were properly diluted with PBS for further ELISA analysis. The assay is performed according to the manufacturer’s instructions and the optical density (OD) was determined at 450 nm in a microtiter plate reader.

### Cell Culture and Proliferation Assay of rhNGF-MPs

TF-1 cell line (ATCC) was grown in RPMI1640 medium (Life Technologies) containing 10% Fetal Bovine Serum (FBS; Hyclone), Penicillin/Streptomycin 50 U/50 μg/ml (Sigma Aldrich) and 5 ng/ml of rhGM-CSF (Gibco) and incubated at 37°C and 5% CO_2_. The potency assay, using TF-1 cell line, was performed to evaluate the ability of the rhNGF released from MPs to stimulate biological activity. Briefly, the proliferation assay was achieved in 96 multi-well plates by incubating 15,000 cells/well, previously starved from rhGM-CSF, in the presence of the following concentration of neurotrophin: in pM 6,760, 2,253, 751, 250, 83.5, 27.8, 9.3, 3.1, 1.0, and 0.3. The final volume per well was 150 μl, consisting in 100 μl cell suspension and 50 μl solution of Reference Standard (RS) or Test Article (TA) at previously described concentrations. Cells were incubated at 37°C, 5% CO_2_ for 48 h. Subsequently, 30 µl of Cell Titer 96® Aqueous One Solution (Promega) was added in each well and cells were incubated again for additional 4 h; finally, the optical density (OD) was determined at 490 nm in a microtiter plate reader. To correctly compare the biological activity of TA to RS, 3 comparison dose-response curves were prepared in each assay plate and performed in 4 different 96 multi well plates. The potency assay was performed using cells at passage 2 (P#2) and passage 3 (P#3). The biological activity (potency) of rhNGF is reported as the percentage of the ratio between EC50 of Reference Standard (RS) and EC50 of Test Article (TA).

### Decellularized Lenticules Engineered With rhNGF-MPs

Decellularized lenticules (0.1% SDS-treated; *n* = 3) were dehydrated for 2 h at 60°C and then incubated at RT in rhNGF-MPs suspension (5 mg in 0.175, 0.9% NaCl). After 5 h of incubation, lenticules were removed from the rhNGF-MPs suspension, washed 10 times in 0.4 ml of 0.9% NaCl and processed for scanning electron microscopy (SEM) and ELISA analysis.

For SEM study (*n* = 3), the specimens were fixed similarly as for TEM analyses with 3.5% glutaraldehyde in 0.1 M sodium cacodylate (NaCaCO) buffer for 1 h and stored at 4°C. Samples were then post-fixed in 2% OsO_4_ in the same buffer, washed in distilled water, dehydrated in increasing ethanol series (50%, 70%, and 100%), immersed in hexamethyldisilazane and dried in a desiccator to air-dry at RT. The dried specimens were then mounted onto SEM stub with double-sided carbon tape add, sputter-coated with gold layers (150Å) and viewed under the Phenom XL microscope at different magnifications (Alfatest, Phenom World).

For ELISA assay (*n* = 3), decellularized lenticules engineered with rhNGF-MPS were lysed using 5 mg/ml collagenase IA in PBS 1X (with Ca^2+/^Mg^2+)^ at 37°C for 30 min. This step was carried out to specifically lyse the lenticule preserving the already released rhNGF (supernatant) and the intact rhNGF-MPs (pellet). The lysed samples were centrifuged for 10 min at 8,000 rpm at 4°C and the supernatant, which contains the rhNGF already released by the microparticles, was collected and immediately stored it at −20° until analysis. The remaining pellet, corresponding to intact MPs released by the lenticule upon lysis, was treated with 0.05 ml of Ethyl Acetate to lyse the PLGA shell of MPs, then 0.100 ml of PBS 1X (with Ca^2+^/Mg^2+^) was added to safely extract the rhNGF. The samples were centrifuged for 5 min at 8,000 rpm and the aqueous phase was collected (pellet phase). The Supernatant and “pellet” were each diluted 1:3 in PBS and analyzed by ELISA assay to detect the concentration of rhNGF.

### *In Vitro* Kinetic Release of rhNGF From Decellularized Lenticules

For evaluation of the *in vitro* release profile of rhNGF, 4 lenticules (110 µm thickness), previously frozen and decellularized (0.1% SDS-treated), were dehydrated for 2 h at 60°C and the next day were engineered with 5 mg of rhNGF-MPs as previously described. The lenticules loaded with rhNGF-MPs were then transferred in 150 µl of PBS in polypropylene LowBind tubes (0.5 ml filling volume) and incubated at 37°C under orbital shaking (80 rpm). The following withdrawal times were defined: t0—t2—4—24 −48 h-t5 - t7-t9 - t12 - t14 - t16 - t19 - t21 - t23 - t25 days. At each time point, each lenticule was picked and transferred into a new vial containing 150 µl of fresh PBS 1X, then placed back in the incubator at 37°C at 80 rpm. The samples collected at each time point, were immediately stored at −20°C for the ELISA assay, in order to determine the rhNGF concentration.

After the t25 daytime point, the lenticules were lysed with 5 mg/ml collagenase IA solution in PBS 1X (with Ca^2+^/Mg^2+^) at 37°C for 1 h and the samples were centrifuged at 8,000 rpm for 10 min in order to collect the MPs. Subsequently, each pellet was lysed with 0.1 ml of ethyl acetate and 0.1 ml of PBS 1X (with Ca^2+^/Mg^2+^) to guarantee the extraction of rhNGF from the intact residual MPs possibly present in the lenticule. After centrifugation at 8,000 rpm for 5 min, the supernatant containing rhNGF was collected. The lysed samples were also stored at −20°C for ELISA analysis.

### Evaluation of the Activity of rhNGF Released From Engineered Decellularized Lenticules

The activity of rhNGF released from engineered lenticules was analyzed by luciferase assay. The test was carried out using rat pheochromocytoma cell line (PC-12) that stably express the human c-fos promoter, activated by rhNGF, driving a luciferase reporter gene (PC12-Luci).

As previously reported, the treatment of PC12-Luci with rhNGF induces the expression of c-fos, monitored by luminescence analysis. The luminescence emission consequently results in rhNGF activity (Milbrandt, 1986).

Briefly, PC12-Luci cells were grown in DMEM F-12 Glutamax medium (GIBCO) supplemented with 5% heat inactivated FBS (Hyclone), 10% heat inactivated horse serum (HS), Penicillin/Streptomycin 50 U/50 μg/ml (Sigma Aldrich) and incubated at 37°C, 5% CO_2_. The cells were grown in CellBind dishes (Corning).

PC12-Luci cells (25000/well) were seeded in complete culture medium in black 96 multiwell plate previously coated with collagen type I (50 μg/ml). After 24 h, cell medium was carefully removed and cells were incubated with rhNGF (released from engineered lenticule and reference standard) in standard tyrode’s buffer with 0.1% BSA for 4 h at 37°C, 5% CO_2_. After the incubation, the basal level of luminescence was monitored for 30 s, by using Enspire multiplate reader (PerkinElmer, PC12-luci NGF 2019). Then, a Triton lysis solution (25 mM Tris, 25 mM Na_2_HPO_4_, 2 mM DTT, 10% Glycerol, 2% Triton X-100) was mixed 1:1 with Luciferin mix (20 mM Tricine, 2.67 mM MgSO_4_, 0.1 mM EDTA, 33.3 mM DTT, 270 µM Coenzyme A, 530 µM ATP and 470 µM Luciferin) was added to cells (50 μl/well) to lyse the cells. The obtained luminescence signal was monitored for then for 60 s. The test was carried out at 0,3750 and 0,1875 ng/ml concentrations.

### Statistical Analysis

All data are expressed as mean ± standard deviation (SD). Statistical analysis was performed using the Mann-Whitney *U* test for DNA quantification, and histological analysis (GraphPad Prism Software). For TEM morphometric quantification a *t*-test or chi-square test was used (GraphPad Prism Software). For Fluo-MPs counts a linear regression analysis was performed with Minitab statistical software. The data obtained from ELISA and potency assay were analyzed using Graphpad Prism software. A *p*-value < 0.05 was considered statistically significant.

## Results

### Decellularization Treatment Efficacy

**FIGURE 1:**
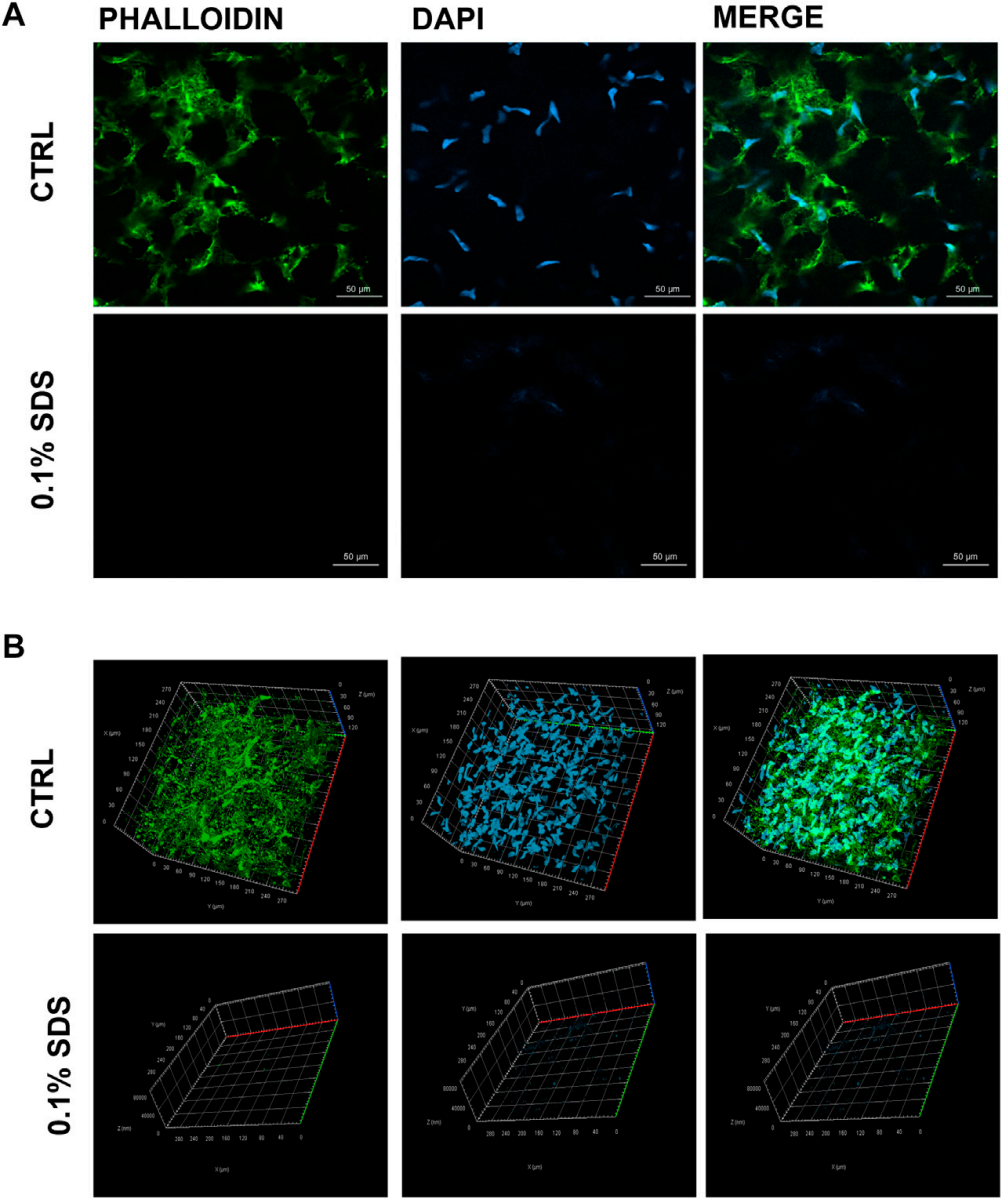
Residual cellular material in decellularized lenticules. Representative **(A)** 2D and **(B)** 3D (Z-stack; *x*, *y,* and *z* axis) immunofluorescence images of lenticules treated or not with SDS (0.1%). Lenticules were directly immunostained *ex vivo* for F-actin (phalloind in green) and nuclei (DAPI in blue) (*n* = 6).

**FIGURE 2:**
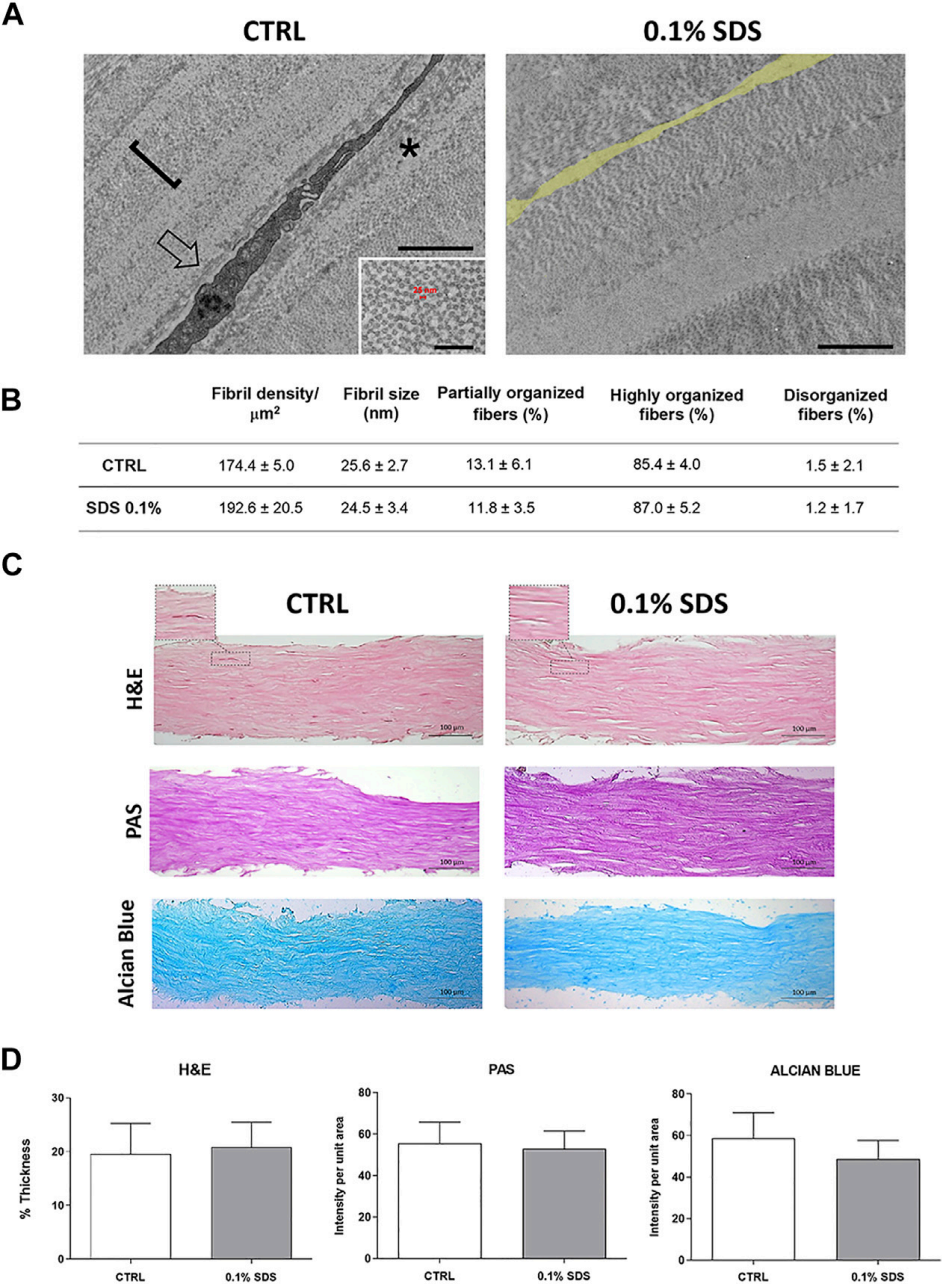
Collagen fibrillar structure and morphology in decellularized lenticules. **(A)** Representative TEM images of lenticules showing a keratocyte (arrow in the left panel) or empty cell space (false labelled in yellow in the right panel), in control and 0.1% SDS-treated lenticules, respectively. Asterisk marks a partially organized collagen fibers. Scale bar: 2 μm (inset, 500 nm). **(B)** Morphometric TEM analysis of collagen fibril size (calculated as indicated in the inset of the TEM left panel), density (one fibril is marked with the square bracket in the TEM picture) and organization in control and 0.1% SDS-treated lenticules. Data are presented as mean ± standard deviation (SD). Significance was assessed using a *t*-test (for fibril density and size) or a chi-square test (for percentages of organized fibers). **(C)** Representative histochemistry images of control and 0.1% SDS-treated lenticules (20x). Lenticules were stained for H&E (Thickness), PAS (Glycoprotein content) and Alcian Blue (Glycosaminoglycans content) (Scale bar 100 μm). AuthorAnonymous, **(D)** Histogram shown lenticules thickness (H&E) and the quantification analysis of PAS (glycoproteins) and Alcian Blue (glycosamminoglycans) staining intensity. Data are presented as mean ± standard deviation (SD) and are compared to untreated condition (*n* = 6).

The efficacy of SDS 0.1% to remove keratocytes from SMILE-derived human stromal lenticules (100-150 µm thickness) was assessed. The 2D and 3D immunofluorescence images (Figure 1) showed in 0.1% SDS-treated lenticules a significant reduction both in phalloidin and DAPI signals compared to untreated lenticules. Particularly, SDS treatment successfully remove cellular material leaving only a minimal residue of nuclear material. However, the DNA extraction and quantification confirmed that the DNA content was significantly lower (*p* < 0.01) in 0.1% SDS-treated lenticules (61.40 ± 11.55 ng/mg) compared to untreated ones (221.7 ± 42.5 ng/mg; Supplementary Figure S1A). In addition, transmittance results confirmed that SDS 0.1% did not induce any significant changes in lenticules’ transparency (Supplementary Figures S1B,C). The immunofluorescence results were corroborated by TEM qualitative analysis which showed keratocytes in control untreated lenticules and empty cell space in 0.1% SDS-treated lenticules (Figure 2A). In addition, TEM morphometric analysis revealed also that decellularization treatment did not induce significant changes in collagen fibrils size and organization as fibril density, fibril size, percentage of partially, highly, and disorganized fibers did not change significantly after decellularization by 0.1% SDS if compared to control samples (Figure 2B).

The effects of 0.1% SDS treatment onECMorganization and content was also assessed. As shown by H&E, PAS, and Alcian Blue stained sections and their relative quantification analyses (Figures 2C,D), lenticules’ thickness and stromal glycoprotein and glycosaminoglycans content were not influenced by 0.1% SDS treatment.

### Analysis of Decellularized Lenticules Engineered With Fluo-MPs

0.1% SDS-treated lenticules were dehydrated (2 h at 60°C) and then incubated with a suspension of Fluo-MPs (5 mg in 0.175 ml of 0.9% NaCl) used as engineering process control.

**FIGURE 3:**
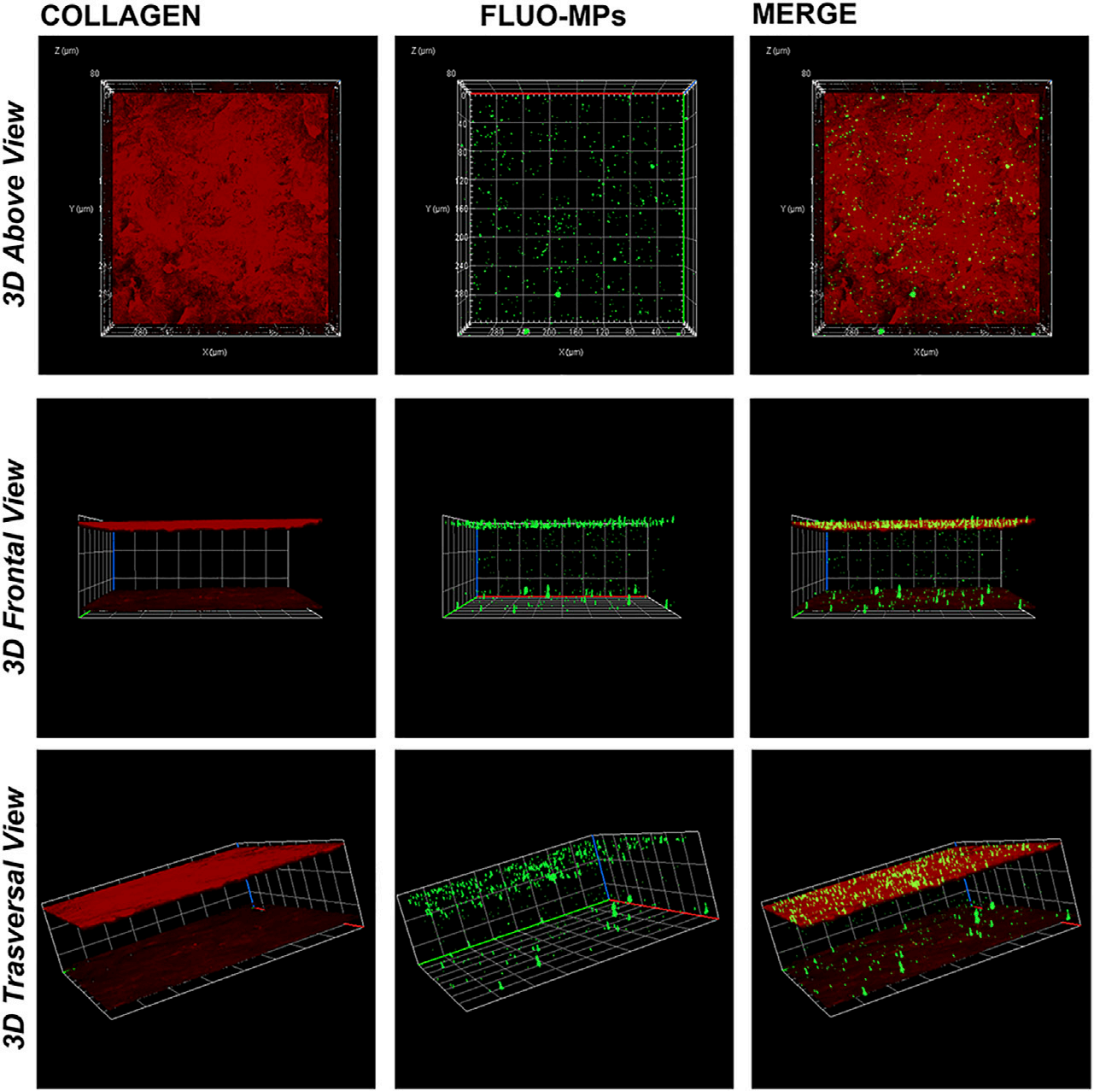
Decellularized lenticule loaded with Fluo-MPs. Representative 3D (Z-stack; *x*, *y* and *z* axis) immunofluorescence images of decellularized lenticules engineered with Fluo-MPs. Lenticules were directly immunostained *ex vivo* following 5 h of incubation with Fluo-MPs suspension. Lenticules upper and lower surface were stained for collagen type I (in red). Fluo-MPs were visualized in green (*n* = 6).

Following different time points incubation (3, 5, 7, and 12 h), immunofluorescence analyses revealed the feasibility to engineer decellularized lenticules with Fluo-MPs which remained immobilized mainly at the lenticules’ surface stained for the Collagen I. Of note, as previously explained in Materials and Methods, through marking out the lenticule’s upper and lower surface, it was possible appreciate the loading of Fluo-MPs also in the depth of the tissue (Figure 3). In addition, a significant correlation between the incubation time and the Fluo-MPs loading yield was found (Supplementary Figure S2). However, since 5 h incubation was sufficient to load an enough number of Fluo-MPs to decellularized lenticules (Figure 3), we chose this incubation time for the subsequent experiments.

### Characterization of PLGA-MPs Loaded With rhNGF

**FIGURE 4:**
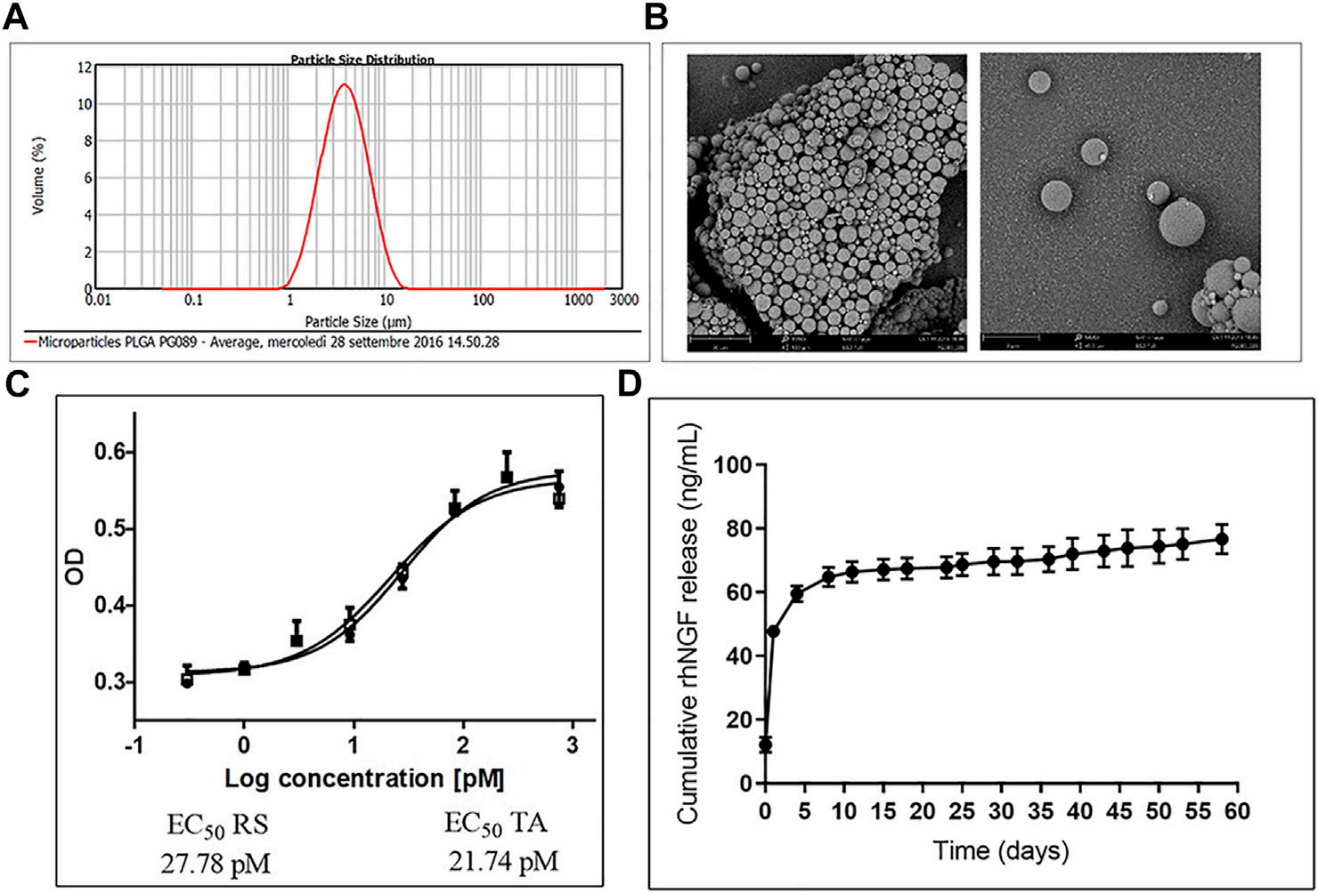
Characterization of rhNGF-MPs. **(A)** Distribution curve showing particles size of rhNGF-MPs between 2 and 6.8 µm, d (0.5) = 3.6 µm, obtained using Mastersizer 2000 (Malvern). **(B)** Example image of dried microparticles obtained using Scanning Electron Microscopy (SEM). The image shows the smooth and spherical shape of MPs coupled with size measurement. **(C)** Representative potency assay graph (optical density at 490 vs. Log concentration in PM). The test confirms the biological activity preservation of rhNGF extracted from MPs (TA, empty square) compared to a reference standard (RS, filled circle). **(D)** *In vitro* release kinetic. The graph shows the release profile (cumulative) of rhNGF from MPs from t_0_ to 60 days.

The produced PLGA-MPs, analyzed *via* ELISA after rhNGF extraction, showed an encapsulation efficiency of about 79% (corresponding to 60 ng rhNGF/mg MPs), with a particle size distribution between 2 and 6.8 µm [d (0.5) = 3.6 µm], as measured with the Hydro 2000 µP wet sample dispersion method using the analyzer Mastersizer 2000 (Malvern) (Figure 4A). Moreover, the MPs visualized via scanning electron microscopy (SEM) showed a spherical shape and a smooth surface (Figure 4B) as desired (Table 1). In addition, the potency assay performed using the rhNGF, previously loaded and extracted from MPs, demonstrated that the biological activity of the protein was preserved during the encapsulation process (Figure 4C). Finally, we checked the release profile of the rhNGF-MPs. The Figure 4D shows the release profile of the rhNGF from the MPs incubated in PBS at 37°C to simulated body temperature. Particularly, the MPs show an initial release burst of the rhNGF during the first day and then constant and sustained release for other 60 days. The total (cumulative) protein released was about 80 ng/ml.

**TABLE 1:** PLGA-MPs acceptance criteria.

Test	Acceptance criteria
Appearance	Clear, colorless solution, practically free from particulate matter
PSD (Particle size distribution)	3 µm ≤ PSD (0.5) ≤ 9 µm
Morphology by SEM	Smooth-surface spherical particles
rhNGF efficiency of encapsulation (%) by ELISA	˃50%
rhNGF release (mg/ml)	Controlled release (≥50 days)
Potency (TF-1 proliferation bioassay)	70%–130% of reference material

### Analysis of Lenticules Engineered rhNGF-MPs

Based on the data obtained with Fluo-MPs, decellularized lenticules were loaded with rhNGF-MPs. The incubation time of 5 h was chosen with the aim to ensure the incorporation of MPs in lenticules and, at the same time to limit the release of rhNGF from MPs in suspension.

**FIGURE 5:**
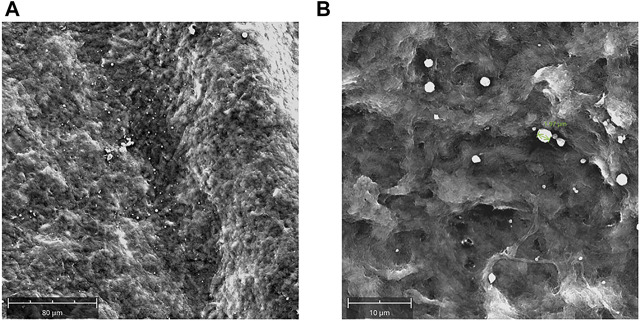
Decellularized lenticules loaded with rhNGF-MPs. Representative SEM images of decellularized lenticules engineered with rhNGF-MPs. Lenticules were fixed in glutaraldehyde (3.5%), dehydrated and sputter-coated with gold layers (150Å). Lenticules surface were captured at different magnifications **(A,B)** by using SEM Phenom XL (Alfatest) (*n* = 3).

The engineering of decellularized lenticules with rhNGF-MPs was confirmed by SEM images (Figure 5). In details, as revealed by representative images of lenticule surface at different magnification, the rhNGF-MPs were loaded to and between collagen fibers of decelllulirized lenticules

In a second step, the lenticules loaded with rhNGF-MPs, were lysed and analyzed by ELISA to verify the presence or not of rhNGFthrough its quantification. Data reported in Table 2 shown that rhNGF-MPs were successfully included in the lenticules. The selective lysis of lenticules allowed extraction of the intact MPs (larger population), still loaded with rhNGF, from the lenticule and to obtain the free rhNGF already released from nanoparticles and small MPs (Table 2, supernatant). Thereafter, the degradation of intact MPs allowed extraction of the remaining amount of rhNGF which was still encapsulated in the PLGA shell (Table 2, pellet phase). As shown in Table 2, the total amount of rhNGF detected was between 4.4 ng/ml and 7.6 ng/ml.

**TABLE 2:** Title evaluation of rhNGF obtained from funtionalized lenticules.

Sample		Concentration rhNGF (ng/ml)	Total rhNGF (ng/ml)	rhNGF distribution (%)
Lenticule 1	Supernatant (Released rhNGF)	6,4	7,6	84
	Pellet	1,2		16
	(Intact MPs)			
Lenticule 2	Supernatant (Released rhNGF)	3,5	4,4	79
	Pellet	0,9		21
	(Intact MPs)			
Lenticule 3	Supernatant (Released rhNGF)	3,9	4,6	84
	Pellet	0,7		16
	(Intact MPs)			

### *In Vitro* Kinetic Release of rhNGF From MPs Included in Decellularized Lenticules

**FIGURE 6:**
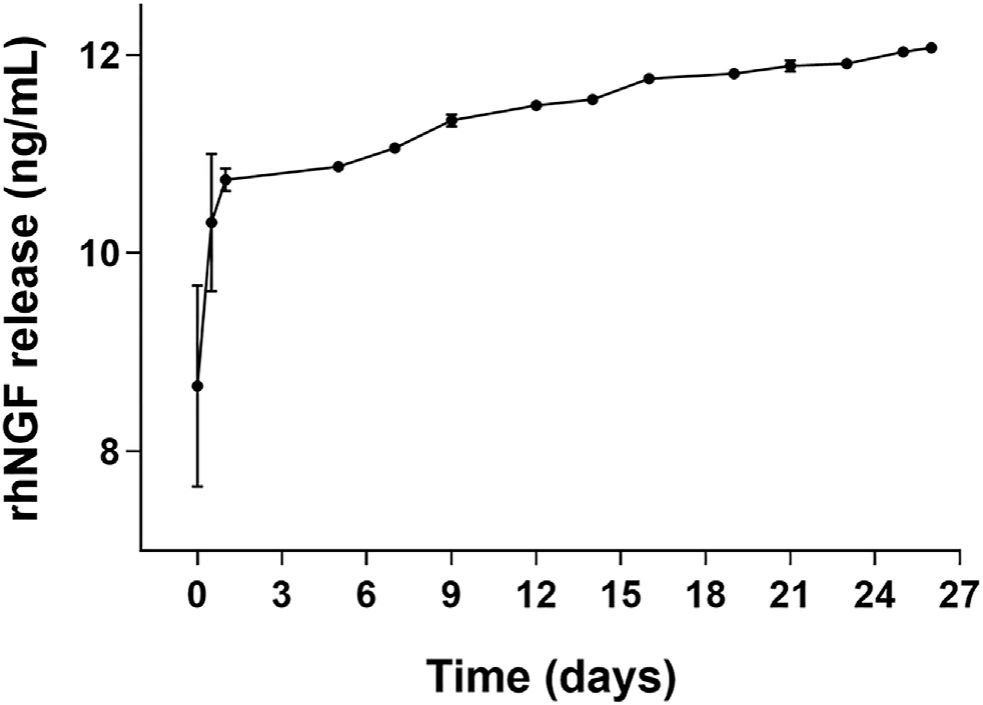
*In vitro* release kinetic of rhNGF-MPs-loaded lenticules. The graph shows the cumulative release of rhNGF from the MPs previously loaded to decellularized lenticule. Samples are collected every 2 or 3 days and the experiment was maintained for 25 days. Data are reported as mean ± SD (*n* = 4).

Experiments of kinetic *in vitro* of rhNGF release from the engineered decellularized lenticules were performed. Figure 6 shows the release profile of rhNGF from the engineered lenticules. The kinetic analyses indicates that rhNGF *in vitro* is rapidly released during the first 24 h (about 80%), with a massive release in the first 2 h and with a slow release sustained for up 25 days.

### Evaluation of the Activity of rhNGF Released From Engineered Decellularized Lenticules

In order to evaluate if the activity of rhNGF released from the MPs previously incorporated in the decellularized lenticules was preserved, the PC12-Luci luciferase assay was carried out.

**FIGURE 7:**
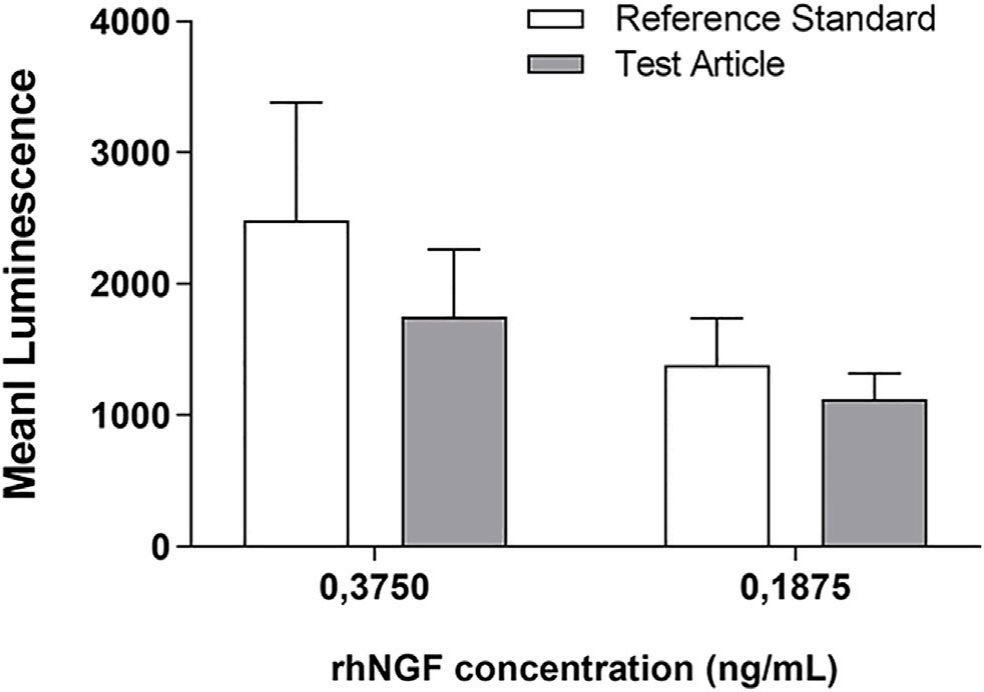
rhNGF activity test using PC12-Luci cell line. The graph shows the luminescence emission of active rhNGF released from the engineered lenticules (Test Article, grey bar) compared with rhNGF reference standard (white bar). The rhNGF reference standard tested was used at 2 different concentrations (0,3750 and 0,1875 ng/ml). Data are reported as mean ± SD (*n* = 3). (Mann-Whitney *U* test; *p* > 0.05, not significant).

The graph reported in the Figure 7 showed that the l activity of rhNGF was preserved during the process of lenticule engineering. Indeed, the results revealed that the activity of released rhNGF is preserved at 70% and 81% compared to the rhNGF reference standard, respectively at 0.3750 and 0.1875 ng/ml concentrations.

## Discussion

SMILE is a minimally invasive surgical procedure which allows correction of myopia through the creation of an intrastromal flap and a peripheral incision by using a FSL (Sekundo et al., 2011; Huang and Melki, 2021). Different to the conventional excimer laser-based refractive surgery, such as the Photorefractive Keratectomy (PRK) and Laser Assisted *in Situ* Keratomileusis (LASIK),in which the process of laser photoablation breaks molecular bonds and vaporizes layers of corneal tissue into atomic constituents (Angunawela et al., 2012), FSL uses ultra-short pulses of light at high repetition rates creating small cavitation bubbles. This establishes an intrastromal cleavage plane with minimal collateral tissue damage (Soong and Malta, 2009).

Corneal stromal lenticules are the discarded tissue obtained following SMILE surgery and, in the recent years, their use in stromal engineering (Wu et al., 2015; Damgaard et al., 2018; Mastropasqua et al., 2018; Nubile et al., 2021) has garnered considerable interest also related to important aspects, including minor ethical issues and the possibility to cryo-store them (Ganesh et al., 2014; Liu et al., 2017) preserving their optical and biomechanical characteristics (Li et al., 2012; Tripathi et al., 2016). In this regard, considering the availability of discarded lenticules deriving from the high number of SMILE procedures performed every year worldwide (Lagali, 2020) it is important to mention the potential of banking lenticules after SMILE (Trias et al., 2020).

On this basis, our study aimed to explore an innovative application of such discarded tissue as natural and biocompatible scaffolds as potential sustained ocular drug deliverysystem. The background to this approach is that, at present, the topical drug administration of eye drops, is the preferred and conventional method for the treatment of most ocular diseases (Jumelle et al., 2020). However, only 5% of the total amount of drug administered is available for a sufficient duration in contact with the ocular surface to exert a therapeutic effect (Ghate and Edelhauser, 2006). Therefore, to improve ocular bioavailability, several alternative delivery approaches have been developed. Among these, are the use of gelling systems such as the fibrin glue, already approved for stem cells delivery (Pellegrini et al., 2018) and drug encapsulation into natural or synthetic micro- and nanoparticles (MPs). These latest techniques enable attainment of a higher concentration at the ocular surface by protecting the active molecule from enzymatic degradation (Patel et al., 2013; Imperiale et al., 2018). In addition to the above, hydrogel and/or collagen based-contact lenses or synthetic scaffolds laden with drugs incorporated in MPs also showed promising results (Maulvi et al., 2016; Sapino et al., 2019; Lynch et al., 2020; Xeroudaki et al., 2020). However, the development of a natural, biocompatible, non-immunogenic scaffold that allow to release an effective and constant concentration of drug molecules for a sufficient period in the corneal target tissues, would be very welcome. In this regard, the use of the biocompatible and biodegradable human amniotic membrane (hAM) to deliver drugs on the ocular surface has been recently investigated (Wan et al., 2021), since hAM is already widely used in the management of a variety of ocular surface conditions (Ramachandran et al., 2019). hAM though has variable transparency and opaque residue. An alternative natural scaffold, with identical structural characteristics as the host cornea, would be more appropriate and possibly more compatible and safer than hAM. For this reason, we herein propose a valid option in the form of FSL prepared corneal stromal lenticules, which are highly reproducible and available in abundance.

The cornea is regarded as an immune privileged tissue and the corneal stroma even more so due to the low immunogenicity of keratocytes (Hori and Niederkorn, 2007). Nevertheless, in this study we decided to use previously decellularized human SMILE derived-stromal lenticules. Even though stromal lenticule re-implantation *in vivo* seems to be a safe approach (Angunawela et al., 2012; Pradhan et al., 2013; Liu et al., 2015; Jacob et al., 2017; Damgaard et al., 2018), graft rejection and inflammation can still occur due to the presence of cellular debris such as lipid membranes and antigens (Hashimoto et al., 2016). To overcome this limitation, several decellularization methods have been tested (Lynch and Ahearne, 2013; Wilson et al., 2016; Huh et al., 2018). The best method, as also demonstrated in our previous study, was an optimized protocol based on SDS detergent which was an efficient approach to remove both cellular and nuclear material from stromal lenticules (Yam et al., 2016). In our initial studies thin lenticules (70 μm thick) from customized human cadaveric cornea were used, while here we used discarded human stromal lenticules directly sculpted and removed from healthy donors undergoing myopic SMILE surgery. These SMILE-derived tissues had a thickness ranged from 100 to 150 µm and despite this variation, the efficiency of 0.1% SDS decellularization treatment, compared to other approaches such 0.1% Triton and 2.5% Trypsin-EDTA (data not shown) was proved. (Figures 1, 2). Of note, this decellularization protocol not only made these lenticules less immune-reactive and more compatible in terms of immunological properties, but also created pores into the scaffold with visible spaces generated among the collagen fibers that could facilitate the intra-tissue incorporation of MPs.

For this reason, we further tested, our novel approach to engineer decellularized human stromal lenticules with a suspension of PLGA-MPs. PLGA is a Food and Drug Administration (FDA)-approved synthetic copolymer of Poly (lactic acid) (PLA) and poly (glycolic acid) (PGA), known for its biodegradability and biocompatibility and is widely used in medical applications (Su et al., 2021). Several advantages have been addressed using PLGA based MPs as nanocarriers for ocular drug delivery (Jumelle et al., 2020). These include protection of encapsulated drugs from rapid inactivation, high encapsulation efficiency and maintenance of slow drug release due to the pace of polymer degradation (Danhier et al., 2012). Hence, PLGA based MPs have already been tested for the treatment of inflammatory ocular diseases (Katara et al., 2017; Gonzalez-Pizarro et al., 2018) and their safety has also been demonstrated both *in vitro* and *in vivo* (Zhang et al., 2018).

Interestingly, in our study we found that the use of an engineering protocol structured into two phases, with a first step of dehydration of the dellularized lenticules, followed by its incubation with PLGA-MPs suspension, with the lamellar expansion occurring in the liquid medium, was particularly advantageous in incorporating PLGA-MPs into the stromal scaffold. Indeed, this optimized approach allowed to homogeneously and rapidly incorporate Fluo-MPs into the decellularized lenticules, through a “soaking-expansion-absorption” phenomenon. The engineering process’s efficiency at different incubation time points was demonstrated by the Fluo-MPs loading both on the surface and in the depth of the stromal tissue (Figure 3, Supplementary Figure S1). These results were also confirmed by using PLGA-MPs containing rhNGF (Figure 5). In detail, spherical-shaped with a smooth surface PLGA-MPs were used to obtain both the effective incorporation into the corneal stromal scaffold and a subsequent adequate release rate of the pharmaceutically active molecules, with an encapsulation efficiency of about active 60 ng rhNGF/mg MPs and a particle size distribution between 2 and 6.8 µm were produced (Figure 4; Table 1). Considering the average dimension of the inter-fiber space generated after the decellularization process, we assumed that the smaller population of rhNGF-MPs (average size of 2–3 µm) and the nanoparticles fraction (average size range 100–500 nm) could be incorporated into the lenticule in-between the collagen fibers. Moreover, it is likely that the mucoadhesive property of PLGA allow a portion of the rhNGF-MPs with greater size, to adhere to the collagen fibers of surface of the lenticules, even after the washing phase of the procedure. Following the engineering process, the total amount of rhNGF detected in decellularized lenticule samples was between 4.4 ng/ml and 7.6 ng/ml (Table 2). It is worth to noting that the variability in the amount of loading of total rhNGF-MPs measured in the lenticules, and consequently the slight variation in the total rhNGF observed, could be due to different thickness of the lenticules used. Interestingly, this bio-scaffold quickly released the pharmaceutically active molecule in the first 24 h followed by a sustained release *in vitro* of rhNGF for up to 1 month (Figure 6). It is important to point out that about 80% of detected rhNGF (initial burst), corresponded to the protein already released from the nanoparticles, small MPs, and from rhNGF attached to the lenticle surface. Whereas the remaining 20% corresponded to the rhNGF still encapsulated into the intact and larger MPs that support the prolonged release. These results are in agreement with those obtained in a recent study conducted by Xeroudaki and colleagues in which they used a synthetic porous collagen-based hydrogel scaffold directly loaded with the NGF (Xeroudaki et al., 2020).

Finally, employing an *in vitro* model PC12-Luci cells, we have also demonstrated that the rhNGF released from loaded lenticules preserves its activity, between 70% and −80% (Figure 7), once compared to a rhNGF reference standard. However, to assess the bioactivity or functionality of rhNGF released from the scaffold further studies will be necessary. Therefore, an *in vivo* pre-clinical study using an animal model of neurotrophic keratitis (keratopathy) is already planned as the next stage of this study. Nevertheless, this is an proof-of concept study which showed the development of processes involved in the incorporation and release of active rhNGFfrom a natural scaffold, suggesting a possible novel application of stromal lenticule as ocular drug delivery system of rhNGF and/or other pharmaceutically active molecules. Among these, antibiotics and anti-Vascular-Endothelial Growth Factor (VEGF) can be similarly deployed to treat infection and vascularization respectively (Bremond-Gignac et al., 2011; Kimoto and Kubota, 2012). Desired shapes, sizes and thickness of lenticules can be constructed from eye bank donor eyes, for example from the donor material remaining after Descemet’s membrane endothelial keratoplasty, making use of precious donor material more efficient. Smaller lenticules can be designed and engineered with anti-VEGF for implantation near the limbus to treat active vascularization; and similarly, such lenticules can be engineered with antibiotics for implantation in small pockets in the vicinity of active infective ulcers. One limitation of using SMILE lenticules for clinical use is the testing of lenticule-donors for transmissible diseases such as HIV, Hepatitis and others which are mandatory for all donor tissues. Eye bank donor tissue is usually tested before release for clinical use. However, both sources can provide a large number of lenticules that can be processed and stored in a desiccated/cyopreserved state and processed for incorporation of the desired molecules before use. This could herald the beginning of a novel mode of ocular drug delivery to treat a variety of corneal pathology.

## Conclusion

Overall, this study demonstrated that stromal lenticules can be efficiently engineered with rhNGF-PLGA-MPs, which remain incorporated in the collagen fibers of this tissue. The extended release of rhNGF for up to a month from the lenticule’s collagen matrix points its possible application as natural, biocompatible, non-immunogenic ocular delivery system able to release active drug.

## Data Availability

The original contributions presented in the study are included in the article/Supplementary Material, further inquiries can be directed to the corresponding author.
