# Supplementary figures and images for: Bioengineered Human Stromal Lenticule for Recombinant Human Nerve Growth Factor Release: A Potential Biocompatible Ocular Drug Delivery System
Source: Front Bioeng Biotechnol. 2022 Jun 23;10:887414. doi: 10.3389/fbioe.2022.887414 (PMC9260024; doi:10.3389/fbioe.2022.887414)

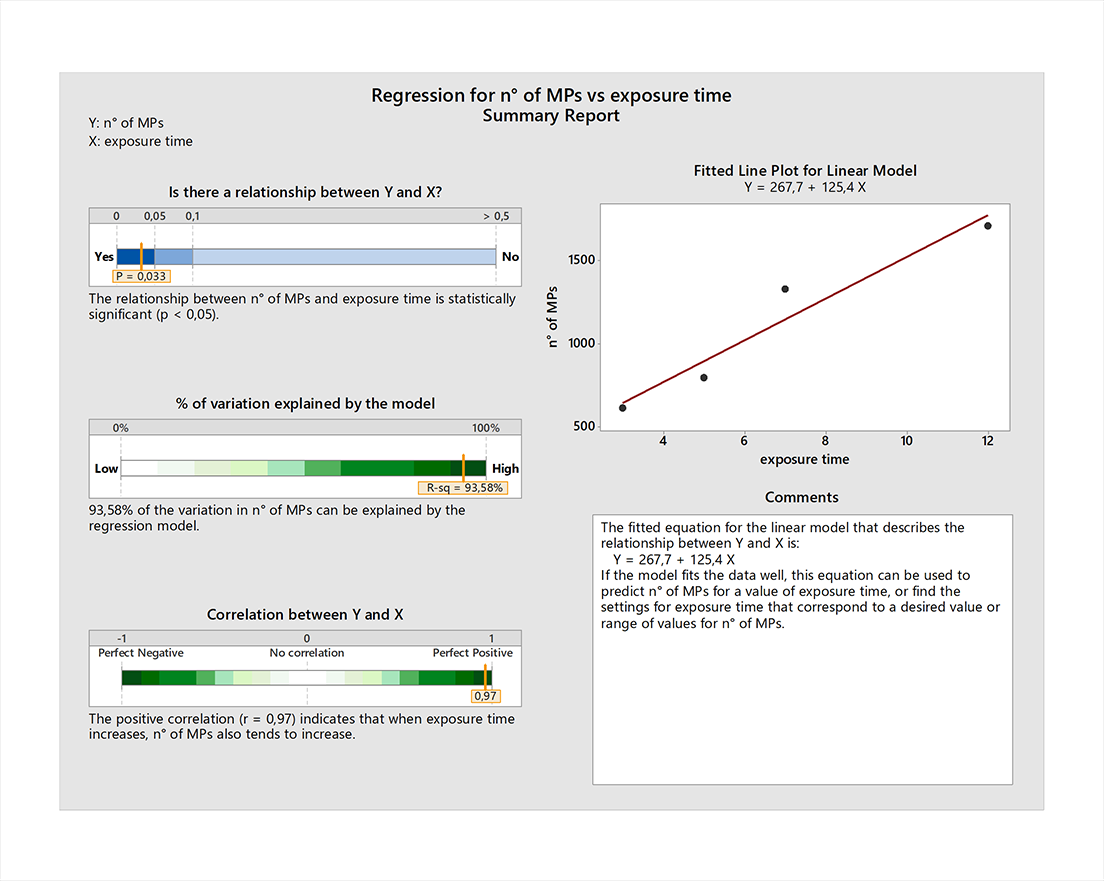

Supplement: Supplementary file 1 [file Image2.TIF]

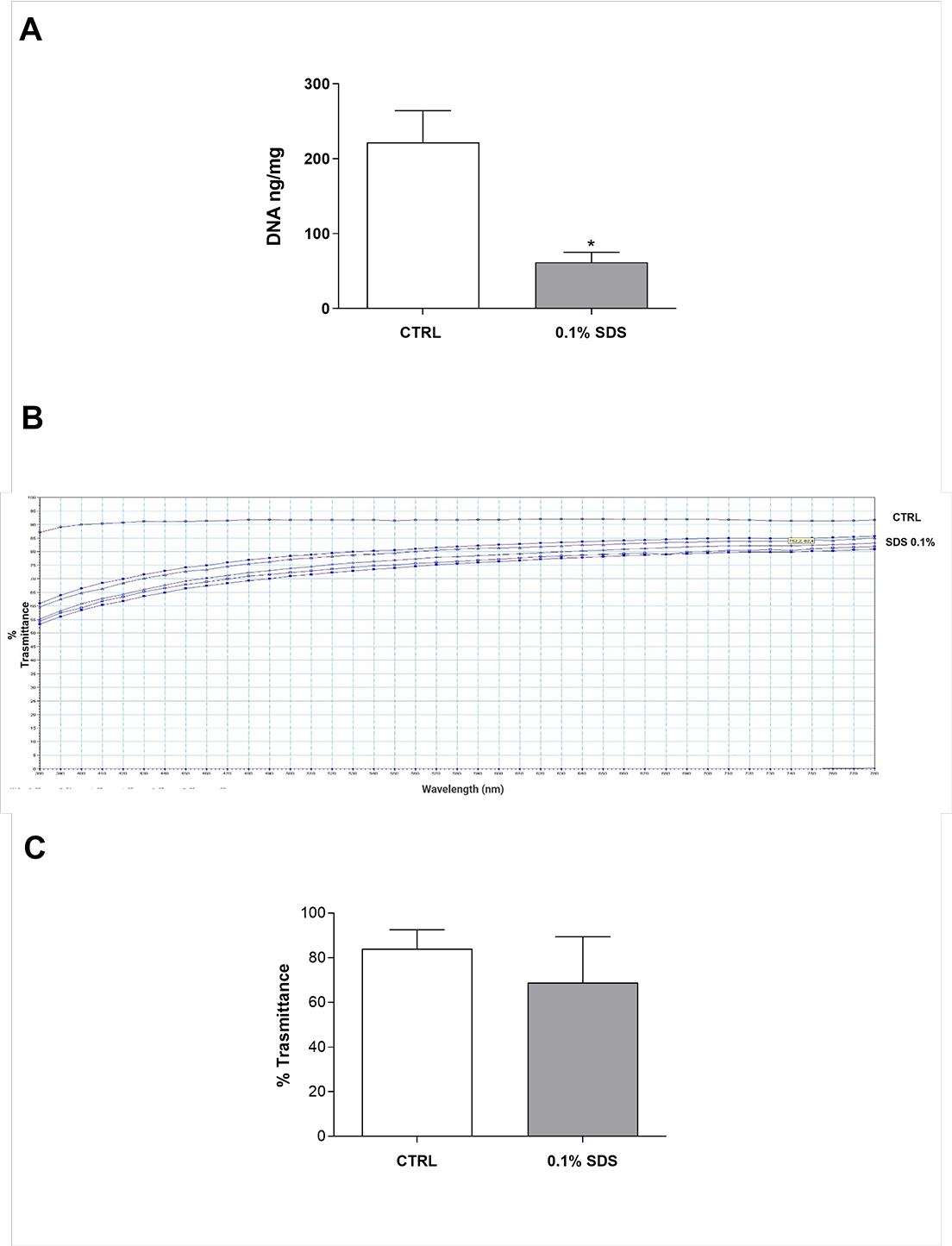

Supplement: Supplementary file 2 [file Image1.TIF]
