# Supplementary material for: Bioengineered Human Stromal Lenticule for Recombinant Human Nerve Growth Factor Release: A Potential Biocompatible Ocular Drug Delivery System
Source: Front Bioeng Biotechnol. 2022 Jun 23;10:887414. doi: 10.3389/fbioe.2022.887414 (PMC9260024; doi:10.3389/fbioe.2022.887414)
Supplement: Supplementary file 3 [file DataSheet1.docx]

**Supplementary Figure Legends**

**Supplementary Fig 1.** **DNA Content and Transmittance.** A. Histograms show the DNA content in control (CTRL) and 0.1% SDS-treated lenticules. Data are reported as mean ± SD (n=6). (Mann-Whitney U test; *p<0.05 vs CTRL). B. Spectral transmittance at wavelengths ranging from 380 to 780 nm. C. The graph shows a similar transmittance between SDS 0.1% treated lenticules *versus* control (CTRL; untreated) ones. Data are reported as mean ± SD (n=5). (Mann-Whitney U test; p>0.05; not significant).

**Supplementary Fig 2. Time incubation and Fluo-MPs inclusion: linear regression analysis.** The scatter plot shows the linear regression function between the time of incubation (exposure time – x axis) and the number of Fluo-MPs loaded (n° of MPs – y axis). Beside to the scatter plot are also reported the statistical parameters that explain such relationship. The regression coefficient suggest a positive linear relationship (R^2^=93.58% with p<0.05) also confirmed by the calculated pearson’s correlation factor (R=0.97). Analysis was performed by using Minitab statistical software.
